# Label-Free Differential Proteomics and Quantification of Exoenzymes from Isolates of the Entomopathogenic Fungus *Beauveria bassiana*

**DOI:** 10.3390/insects7040054

**Published:** 2016-10-14

**Authors:** Giuseppe Dionisio, Per Kryger, Tove Steenberg

**Affiliations:** 1 Department of Molecular Biology, Aarhus University, Flakkebjerg, Forsøgsvej 1, 4200 Slagelse, Denmark; 2 Department of Agroecology—Entomology and Plant Pathology, Aarhus University, Flakkebjerg, Forsøgsvej 1, 4200 Slagelse, Denmark; per.kryger@agro.au.dk (P.K.); tove.steenberg@agro.au.dk (T.S.)

**Keywords:** *Beauveria bassiana*, differential proteomics, Progenesis QI for proteomics, exoenzymes, subtilisin protease, chitinases, cockroach, cuticle-degrading serine proteinase

## Abstract

*Beauveria bassiana* is an entomopathogenic fungus that grows both in vivo and in vitro. In vivo it can colonize live insect hosts, and tissue digestion occurs by secreted hydrolytic exoenzymes. It can also colonize dead insect tissue provided this is free from competing microorganisms. Depending on whether the host is alive or dead the expression (quality/quantity) of the exoenzymes may vary. We have grown several isolates of *B. bassiana* in shaking flasks for 120 h at 25 °C in order to evaluate the maximal exoenzyme production using two diet regimes. As sole carbon, nitrogen, and phosphate sources we used 1% shrimp chitin and either 0.5% *w*/*v* of dead intact American cockroach (*Periplaneta americana*) or their isolated cuticles. This is the first report of a differential proteomics of *B. bassiana* exoenzymes performed by label-free nano-LC MS/MS. Total proteolytic enzyme activity was mainly due to Pr1A or Pr1B depending on the isolate and the diet regime. The most differentially secreted enzymes were: the cuticle-degrading subtilisin Pr1A, GH13 alpha-glycosidase, glucan endo-1,3-beta-glucosidase, subtilisin-like proteinase Spm1, lipase 1, beta-1,3 exoglucanase, and endo-1,3-beta-glucosidase. Among the *B. bassiana* isolates analyzed, Bb 678 and Bb BG were the most active in Pr1A secretion.

## 1. Introduction

*Beauveria bassiana* (Hyphocreales, Ascomycota) is a facultative saprophytic fungus with capacity to infect and kill insects and other arthropods and also to colonize a range of plant species as endophytes [1]. Insects are infected via cuticular penetration as fungal spores germinate on the cuticle of hosts. Germ tubes grow through the layers of the cuticle and epidermis using a combination of physical force and enzymatic action and ultimately enter the haemocoel. The fungus can also utilize other carbon sources (e.g., artificial growth media or dead insects free from competing microorganisms). In order to penetrate and invade the host exoskeleton it has developed mechanisms for penetrating both the waxy layer of the epicuticle and the other layers of the integument (chitin, proteins, enzyme inhibitors, and insect components) [2,3,4,5]. Endogenous inhibitors are embedded into the integument [5] among layers of melanin deposition, and cross-linked proteins contribute to the strength of the cuticle in, for instance, cockroaches [6]. Proteins (constituting up to 45% of dry weight, DW) followed by chitin (up to 35% of DW) and lipids (up to 18% of DW) are the predominant matrix components of the cuticle in the American cockroach (*Periplaneta americana*) [7]. Most entomopathogenic fungi (EPF) mainly secrete proteases and, to a lower degree, chitinases or lipases in order to degrade insect cuticles, as cuticular chitin is shielded by proteins [8]. Fungal chitinases (*N*-acetyl-β-glucosaminidases) from entomopathogens showed no detectable activity against insect cuticle either alone or in combination with protease or other chitin-degrading enzymes (chitin esterases, chitosanases, etc.) [8]. The first layer of intact epicuticle to which the fungus propagule attaches contains lipids and wax layers esterified among each other and covalently bound with underlaying layers of protein matrix of the procuticle rich in melanin, a protein rich in cysteines. Some of these are involved in sclerotinization of the procuticle by forming a tight network due to disulfide bridges. Aspecific lipases/esterases are often produced by entomopathogenic fungi during the first step of the process of penetrating the arthropod epicuticle [9,10], meaning that the mode of action of the *B. bassiana* exoenzymes is sequential as previously established by macroscopic observation of the cuticle [11]. To date, different studies have been conducted to identify and quantify the level of exoenzymes from entomopathogenic fungi (inducible or not) and to correlate those enzyme levels to fungus virulence [12,13,14]. Here we present the first proteomics approach for *B. bassiana* isolates in order to establish qualitative and quantitative aspects of secreted hydrolases, including proteases, chitinases, lipases, and phosphates a.o. during aerobic liquid growth using intact cockroaches versus isolated cuticles as sole N/C/P sources. By increasing the information on the secretome of *B. bassiana*, the use of this fungus as a control agent against insect pests such as *P. americana* can potentially be supplemented by *B. bassiana* culture supernatant coming from aerobic fermentation or by using some of the listed exoenzymes discovered here as potential recombinant candidates for a fungus-free control approach, where appropriate. In addition, other uses of *B. bassiana* exoenzymes can be envisioned, for example, in various biotechnological applications such as recombinant lipases to be used in biodiesel production [15].

About 200 species of entomopathogenic fungi from a range of genera have been described and many species are effective biocontrol agents [16]. At present, their genome availability is restricted mainly to *Metarhizium* sp., *Cordyceps militaris*, *Ophiocordyceps sinensis*, and *Beauveria* sp. for OMICS studies [17,18].

Here we aim at using a novel and up-to-date approach to study the proteomics of exoenzymes from *B. bassiana* isolates. This has potential as a tool for identifying important exoenzymes which maybe at a later stage can be introduced as recombinant into other *B. bassiana* isolates in order to improve their performance or produced in vitro as recombinant (i.e., in *Pichia pastoris*) in order to be used as a fungus-free bio-insecticide mixture.

## 2. Experimental Section

### 2.1. B. bassiana Isolation and Maintenance

Six *B. bassiana* isolates were used: Bb 715, isolated from *Stomoxys calcitrans* (Diptera: Muscidae); Bb 678, isolated from *Musca domestica* (Diptera: Muscidae); Bb BG, re-isolated from the commercial product BotaniGard 22 WP^®^ (Laverlam International Corporation (LVM), Butte, MT, USA), originally isolated from *Diabrotica undecimpunctata* (Coleoptera: Chrysomelidae); Bb 813, isolated from *Varroa destructor* (Acari: Varroidae); Bb 949, isolated from *V. destructor*; and Bb 893, isolated from *V. destructor*. All isolates except Bb BG originated in Denmark and were kept stored in glycerol at −80 °C (Aarhus University, Department of Agroecology).

Assignment to *B. bassiana* was based both on microscopic features and molecular identification using PCR on fungal genomic DNA extracted by the CTAB method [19], targeting the ITS (Internal Transcribed Spacer) of the fungal rDNA gene using the primers ITS1 (5′-TCCGTAGGTGAACCTGCGG-3′) and ITS4 (5′-TCCTCCGCTTATTGATATGC-3′) and by cloning the PCR product in pCR4 blunt Topo. Sequencing was performed by GATC Biotech (Cologne, Germany). 

Fungal isolates were subcultured on 1% shrimp chitin agar (C7170, Sigma-Aldrich Chemie Gmbh, Munich, Germany) before growth in liquid media (see Section 2.2.) containing either dead intact cockroach diet (*P. americana*, from an insect rearing) or isolated cuticle diet from crushed cockroaches. Isolated cuticles were prepared by crushing dead cockroaches with mortar and pestle in MilliQ water and filtering the homogenate through three layers of Miracloth (Merch-Millipore, Darmstadt, Germany). The filtrate was centrifuged at 4000× *g* for 10 min and the pellet was washed twice with distilled water and centrifuged again as described above. Subsequently, the liquid media containing either 1% shrimp chitin with 0.5% *w*/*v* intact cockroaches or 0.5% *w*/*v* of isolated cuticle were sterilized by autoclaving. 

### 2.2. Liquid Growth

Fungal isolates were sub-cultured on 1% shrimp chitin agar at 25 °C for six days. Conidia were harvested by adding an aqueous solution of 0.01% Tween 80 and filtering through sterile absorbent cotton. One mL of spore suspension containing a concentration of 3 × 10^8^ spores/mL was added to 250 mL Erlenmeyer baffled flasks containing 50 mL of sterilized shrimp chitin/cockroach medium: 1% shrimp chitin, 0.5% *w*/*v* cockroach-isolated cuticle or 1% shrimp chitin, and 0.5% *w*/*v* intact cockroach as described above. Shaking was performed at 25 °C on a rotary shaker at 200 rpm for six days. One milliliter of media per sample was collected and the supernatant obtained by centrifugation every 24 h during six days, and the final mycelia wet weight recorded. The supernatant was filter sterilized through a 0.2 µm syringe filter (Sartorius) and used for enzyme assay or concentrated 10 times by Vivaspin 15 (Sartorius) centrifugal concentrators (MW Cut Off of 3000 Dalton). The fungus did not grow much more rapidly in the media containing isolated cuticles as compared with the intact insect diet. In both cases the wet weight at the fourth day was comparable (1.2 ± 0.15 g/50 mL media). Considering that the initial pellet containing chitin and cuticle for 50 mL media used was 0.9 ± 0.12 g/50 mL media, the net wet weight recorded at day four was around 0.3 ± 0.14 g/50 mL media. All experiments were performed in triplicate.

### 2.3. Enzyme Activity

We focused on protease activity that was assayed using two chromogenic substrates (from Bachem) for trypsin and chymotrypsin-like activities: Z-Phe-Arg-pNA (carboxy-benzoxy-*N*-Phenylanaline-Arginine-p-nitro-anilide) and Suc-Ala-Ala-Pro-Phe-pNA (Succinil-Alanine-Alanine-Proline-Phenylalanine-p-nitro-anilide). The assay consisted of incubating 10 µL of culture supernatant for 5 min at 37 °C in the presence of 0.1 mM of the chromogenic synthetic peptides as substrate in 0.4 mL of 0.1 M Tris-HCl pH 8.0. The reaction was terminated by adding 0.2 mL of 1.5 M HCl followed by diazotization by 0.1 mL of 0.1% NaNO_2_, 0.1 mL of 1% ammonium-sulfamate, and 0.1 mL of 0.1% of NNED (*N*-(1-Naphthyl)ethylenediamine dihydrochloride), respectively [20] (all reagents from Sigma-Aldrich Chemie Gmbh, Munich, Germany). The color development was stable within 2 h and absorbance at 540 nm was recorded in a microplate reader (Epoch, BioTek Instruments, Inc., Winooski, VT, USA) using the appropriate wavelength filter. Protein concentration was measured using the Bradford assay [21]. Enzyme activities were recorded measuring their absorbance at 540 nm and relating it to the diazotized pNA standard curve. Specific activities (U·mg^−1^·min^−1^) were calculated by dividing enzymes units (U·mL^−1^·min^−1^) to their protein concentrations (mg/mL). Assays were performed in triplicate and standard deviation calculated. 

### 2.4. Proteomics Sample Preparation

Supernatants from day four of liquid cultures of *B. bassiana* (see Section 2.2) were concentrated by Vivaspin 15. Subsequently, 200 μL was denatured and disulfide bonds were reduced using 200 μL of 8 M Guanidine-HCl, 20 μL of 1 M DTT, and 50 μL of 1 M ABC (NH_4_HCO_3_, ammonium bicarbonate buffer pH 8.0) at 56 °C for 30 min. Cysteine alkylation was performed by adding 30 μL of 0.5 M iodoacetamide in 0.1 M ABC buffer and vortexed briefly before incubating for 45 min at room temperature (RT) in the dark. Desalting of alkylated proteins was performed using Vivaspin 500 (MWCO 3000) and the buffer was exchanged as well into 0.1 M ABC buffer.

Hundred μL of concentrated and alkylated proteins were digested with 1 μg of proteomics grade trypsin (Pierce, Thermo Scientific, Waltham, MA, USA). The digestion was performed at 37 °C overnight and then stopped by adding 2 μL of 50% formic acid (FA). To the undesalted peptides 100 fmol/mL final concentration of tryptic digest yeast Enolase 1 was spiked in. Sample preparation was performed in triplicate.

Per sample, 4 µL of desalted peptides were analyzed also in triplicate onto an Xbridge BEH130 C18 5 μm desalting/trap column on-line with a BEH300 C18 1.7 μm nanoUPLC analytical capillary column (100 μm × 100 mm) on an Acquity nanoUPLC-LC system interfaced with a nano source to a Q-TOF Premiere MS (Waters, Milford, MA, USA). The entire length of the LC run was 66 min starting with a gradient of ACN/0.1 FA (0 to 40%) from 0 to 50 min followed by 90% ACN wash and re-equilibration. Data acquisition was performed in V positive mode with Glu-Fib as calibrant (m/z 785.8426) and lock mass. MS and MS/MS data were recorded in MSe mode (MS1 scan every 1.5 s at 10,000 FWH resolution and MS/MS fragmentation of all ions every 1.5 s). MSe runs were also analyzed by Protein Lynx Global Server 2.5 software for validation of protein hits and peptide coverage map. 

### 2.5. Differential Label-Free Quantitative and Qualitative Proteomics

Progenesis QI for Proteomics ver. 2.0 (Nonlinear Dynamics, Newcastle, UK) was used to analyze the different MSe runs in triplicate. Samples were compared according to their exact mass versus retention time ratio after normalization with 100 fmol/µL of yeast Enolase 1 (Uniprot P00924, ENO1, *Saccharomyces cerevisiae*) standard tryptic digest (MASSprep Enolase, Waters, Milford, MA, USA). Peptides were identified using the Progenesis QI for proteomics internal MSe search engines based on PLGS 3.0 and using *B. bassiana* proteins FASTA databases created according to the respective Uniprot sequences. The quantification in Progenesis was performed by the built-in Hi-3 algorithm from Waters which allowed, after Apex 3D peptide identification of the reference protein digest, the quantification of any protein based on their three most abundant peptides. The fixed modification was the carbamidomethyl cysteine (ΔMass +57.02), and other variable modifications were also accounted for in the search, such as: deamination NQ (+0.98), oxidation MHW (15.99), carbamilation N-term (+43.01), dehydration DSTY, C-term (−18.01), hydroxylation DKNPRY (+15.99), etc. The protein normalization method chosen in Progenesis, as mentioned previously, was performed according to ENO1 as well as the absolute quantification. ANOVA statistics were made by the built-in Progenesis statistics module. Protein identification having *p*-value(s) > 0.05 was not included in the analysis.

## 3. Results

### 3.1. Protease Activity Measurement

**Figure 1:**
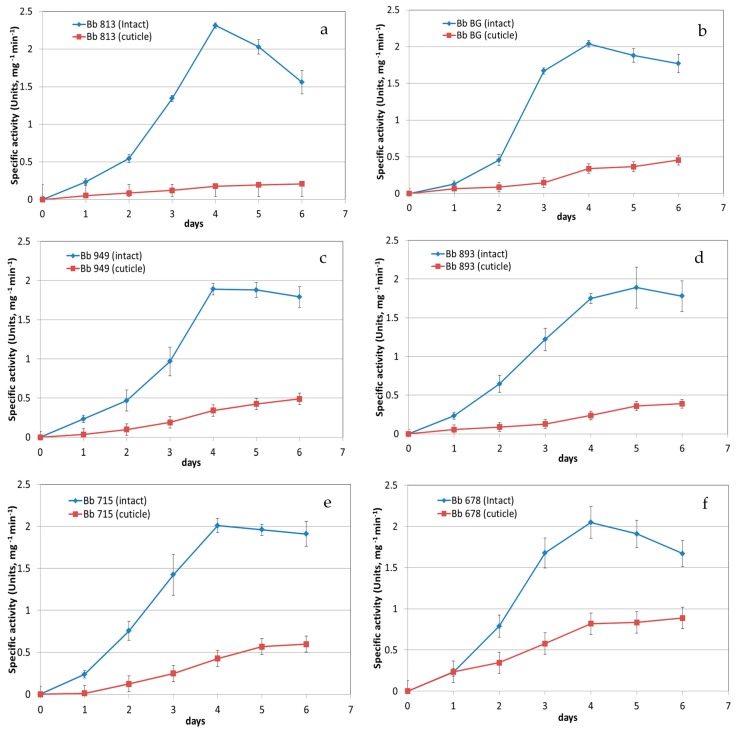
Exoenzymatic activity over time of six *B. bassiana* isolates assayed with Suc-Ala-Ala-Pro-Phe-pNA for detecting chymotrypsin-like activity in liquid shaking cultures using both the intact insect and the isolated cuticle diet medias indicated. Proteolytic curves were obtained for Bb813 (**a**), Bb BG (**b**), Bb 949 (**c**), Bb 893 (**d**), Bb 715 (**e**) and Bb 678 (**f**). Standard deviations has been calculated from triplicate experiments in triplicate technical repeats.

Chymotrypsin-like activity was recorded by the synthetic substrate Suc-Ala-Ala-Pro-Phe-pNA which is used for detecting subtilisin-like proteases. Trypsin-like activity was assayed by Z-Phe-Arg-pNA and as for Suc-Ala-Ala-Pro-Phe-pNA detection was augmented by their diazotisation. Activity measurement was recorded over six days of growth and plateau conditions were most frequently achieved after four days (Figure 1). No trypsin activity was detected over the period in analysis. The peak of the chymotrypsin-like activity was detected at day four (Figure 1). Over six days *B. bassiana* isolates produced the highest chymotrypsin-like activity when cultivated in the medium containing intact cockroaches (Figure 1). In particular, Bb BG and Bb 678 showed an increase of chymotrypsin-like activity of about 7.2 and 6.5 folds higher, respectively, than when grown in a medium containing isolated cuticles only (Figure 1). 

### 3.2. Proteomics Identification of B. bassiana Exoenzymes

To identify the composition of enzymes produced by *B. bassiana* isolates through LC-MS-based proteomics we used isolate 813 cultured in 1% shrimp chitin and 0.5% *w*/*v* of intact cockroaches as a model sample because this isolate has the highest chymotrypsin-like activity when compared to the other isolates under the same conditions (Figure 1). Non-quantitative identification of proteins present after four days of liquid culturing of this isolate is presented in Table 1. The highest number of peptides was detected for the identified proteases Pr1A, an alkaline serine protease (uniprot A0A0A2W0R3, also annotated as a cuticle-degrading enzyme from Genbank) and Pr1B Alkaline protease (A0A0A2VW01), both belonging to the subtilisin family having chymotrypsin-like serine proteases. Other proteases included subtilisin-like proteinase Spm1 (A0A0A2VSY1), two aspartic proteases (A0A0A2V8V1 and A0A0A2WIE5), two carboxypeptidases (A0A0A2VR76 and A0A0A2VKM6), tripeptidyl peptidase (A0A0A2W0E3), and a prolyl-X carboxypeptidase (A0A0A2VGF4). Furthermore, three GH18 chitin-degrading enzymes were detected: Endo chitosanase (A0A0A2VUK), Chitinase D (A0A0A2VG14), and another chitinase (A0A0A2V532). Many glycosidases possibly involved in the participation of integument digestion were also found (A0A0A2VJ73, A0A0A2VDM1, A0A0A2V7C2, A0A0A2VHX5, A0A0A2VL54, A0A0A2V6S0, A0A0A2VB74, A0A0A2VDW0, A0A0A2VWX1, A0A0A2VZ64, A0A0A2VM88, and A0A0A2W3A3). A lipase (A0A0A2W0S0) and a neutral cholesterol ester hydrolase (A0A0A2VCZ9) were found soluble already in the supernatant without Triton X-100 solubilization and may be synergistically involved in the hydrolysis of the epicuticle. Finally, redox exoenzymes that could be involved in the degradation of the cuticle by helping in de-cross-linking as part of a de-melanization system were also detected (A0A0A2VRL8, A0A0A2 V928, and A0A0A2VFU8, Table 1).

### 3.3. Qualitative and Quantitative Label-Free Differential Proteomics of B. bassiana Exoenzymes among the Five Isolates

To quantify the contribution of the different exoenzymes/proteins present in the extracellular supernatant of different *B. bassiana* isolates we performed a qualitative/quantitative differential proteomics based on LC-MS/MS and Progenesis QI for proteomics analysis.

**Figure 2:**
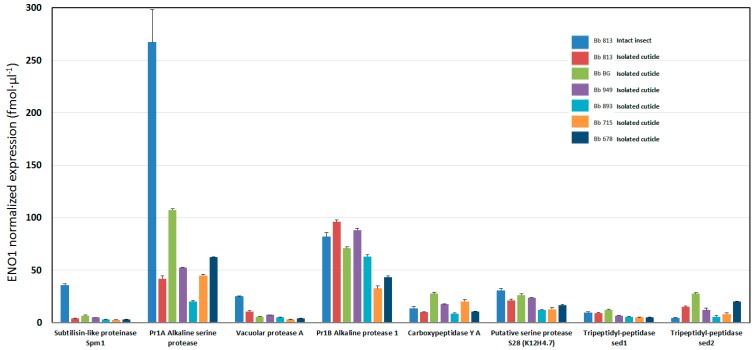
Quantitative label-free proteomics expression of *B. bassiana* proteolytic exoenzymes detected in different isolates in liquid supernatants after four days of liquid shaking culturing. Isolate Bb 813 was evaluated both in intact and isolated cuticle diet, the other isolates were grown as indicated in the X-axis legend. Uniprot accession numbers for these proteases are listed in Table 2. Means and standard deviations are based on triplicate biological experiments, data were ENO1 normalized and recorded in triplicate technical repeats.

The most abundant exo-enzymes expressed after four days were Pr1A (A0A0A2W0R3), Pr1B (another alkaline protease, A0A0A2VW01), and a putative fungistatic producing metabolite oxidase (A0A0A2VRL8) judging from their number of peptides found and quantitative spectral counting and ENO1 normalized (Table 2). Other abundant enzymes were found differentially expressed in our samples as, for example, the Chitinase D (A0A0A2VG14), which was expressed independently of whether the cockroach added was either as an intact cadaver or an isolated cuticle (Table 2). Other chitinases were also found (A0A0A2VUK5 and A0A0A2V532), but those were mainly found in Bb 813 (Table 1 and Table 2). Among the proteolytic set of exoenzymes, a detailed overview of their quantitative expression is shown in Figure 2. In this case the subtilisin-like protease Spm1 and the alkaline protease Pr1A were highest on the intact cockroach diet, thus confirming the activity measurements of Figure 1 based on the total chymotrypsin-like activity of the supernatant. Hence it is clear that the large majority of *B. bassiana* exo-proteases are represented by subtilisin-like proteases (Table 2 and Figure 2). Other glycosidases possibly involved in the participation of integument digestion were also found differentially expressed among the isolates and conditions as shown in Table 2 (i.e., alpha-galactosidase (A0A0A2VJ73), beta-galactosidase (A0A0A2VNH3), beta-glucosidase (A0A0A2W3A3), aldono-lactonase (A0A0A2VPJ3), and sialidase (A0A0A2W9X8). Some endo-1-3-beta-glucosidases were more expressed whenever the fungus diet consisted of intact cockroach (A0A0A2VL34 and A0A0A2VAM6). The exo-beta-1-3 glucanase (A0A0A2V7C2) and the alpha-glycosidase GH13 (A0A0A2VHX5) were also influenced by whether the diet consisted of intact insect or isolated cuticle but varied as well among isolates (Table 2). Exoenzymes not influenced by the substrate (constitutive enzymes) shown in Table 2 were: alkaline phosphatase H (A0A0A2VWS4), glucan 1,3-beta-glucosidase (A0A0A2VZ64), tripeptidyl peptidase sed1 (A0A0A2VAJ1), autolysin (LysM domain-containing protein), catalase-peroxidase (A0A0A2V928), thio-redoxin reductase (A0A0A2VFU8), glutathione reductase (A0A0A2VC64), and alpha-N-arabinofuranosidase (A0A0A2VWX1) (Table 2). In addition, the strongest induced exoenzymes upon intactness of the cockroach were: lipase 1 (A0A0A2W0S0), of which the catalytic activity has not yet been characterized, and isochorismatase-like protein (A0A0A2VIV9), an hydrolase that does not have protease activity but rather hydrolyzes isochorismate to pyruvate and 2,3 dihydro-2,3 dihydroxybenzoate (Pfam PF00857).

## 4. Discussion

This is the first proteomics study for *B. bassiana* exoenzymes and focuses on the effect of intact insects versus isolated cuticles (of the American cockroach) as nutrition and sole N/C/P source for *B. bassiana*. The main objective was to identify and quantify exoenzymes of *B. bassiana* from supernatants when cultivated under these two diet regimes. A range of *B. bassiana* isolates from different hosts were studied to evaluate the qualitative/quantitative nature of their secretome in liquid cultures. The highest total chymotrypsin-like proteolytic activity was observed for the time point at day four for all isolates (Figure 1). For isolate 813 and for the intact diet regime we did a preliminary qualitative and quantitative proteomics (based on spectral counting), which represents a snapshot of the possible exoenzymes at day four. The top two expressed exoenzymes were chymotrypsin-like enzymes (Table 1). In order to verify if those data might suggest either a bias among the isolates or between their diet regimes, we decided to perform a label-free absolute differential proteomics experiment using a common diet regime (isolated cuticle diet) for all the isolates. Additionally, we included isolate Bb 813 with intact insect diet as control in our differential proteomics studies since it showed the highest chymotrypsin-like activity when intact insect diet was used (Figure 1). 

Our qualitative and quantitative data on *B. bassiana* exoenzymes revealed that the predominant proteolytic activity is composed of several proteases belonging mainly to the chymotrypsin-like family (Pr1B and Pr1A, Table 1 and Table 2). Their relative amount also differs among the species analyzed. The Pr1A was mainly secreted upon intact insect diet regime (Table 2). Furthermore, lipases and chitinases were also differentially expressed, being secreted in higher amount and hence accumulating in the case of the intact insect diet versus isolated cuticle (Table 2).

The entomopathogenic fungi *M. anisopliae* and *B. bassiana* display a range of secreted enzyme activities produced as mainly secreted proteases assayed with casein as substrate or chromogenic substrates based on synthetic substrates [22,23]. Recently, transcriptomics and proteomics analysis of the exoenzymes of *M. anisopliae* were published [13,24,25]. The proteomics studies described a time course secretion of exoenzymes (secretome) identified using growth in liquid culture with cuticles from *Dysdercus peruvianum* or macerated *Callosobruchus maculatus*, respectively [13,25]. Compared to the *Metarhizium* sp. secretome [13], our study on *B. bassiana* revealed, at time point 96 h, some overlapping in the secretion of exoenzymes also seen in *Metarhizium* sp. at 48 h. Whereas in *Metarhizium* sp. the main abundant protein secreted is a subtilisin-like protease Pr1B, with Pr1A being 50% of the expression when compared to Pr1B, we found our *B. bassiana* isolates to exhibit different expression levels of Pr1B and Pr1A, depending on the isolate being based on the analysis of a fixed diet nutrition (i.e., intact cuticles, Figure 2). Isolates Bb GB, Bb 715, and Bb 897 secreted the isoform Pr1A more than Pr1B (Figure 2) in contrast to isolates Bb 813, Bb 949, and Bb 893 that secreted relatively more Pr1B (Figure 2). However, if the nutrition consisted of intact cockroaches (only relevant for Bb 813), only Pr1A was over-secreted in the culture medium, while the Pr1B levels remained almost unchanged, as compared to the isolated cuticle diet (Figure 2). The absolute amounts detected for the subtilisin-like exoenzymes in Figure 2 were compared to the total chymotrypsin-like activity present in Table 1 with the conclusion that mainly Pr1A or Pr1B is responsible for such total proteolytic activity, whereas in the case of intactness of the host as nutrition only the Pr1A could be responsible for the increase of the total chymotrypsin-like activity. It is known that a cuticle-degrading protease (CDEP-1, identical to Pr1A) of *B. bassiana* when made as recombinant in *Pichia pastoris* increases the fungal virulence when applied against aphids [26]. In addition, when *M. anisopliae* was transformed to contain more copies of Pr1A, an increase of the virulence compared to the untransformed isolate was observed [27]. Furthermore, in *Metarhizium* sp., a repression of Pr1A gene expression was found when the fungus was grown on dead insects compared to live hosts [28]. Even in some *B. bassiana* isolates overexpression of a subtilisin-like protease (Pr1A) and/or a chitinase (Bbchit1) resulted in an increased virulence [29]. Likewise, the EPF *Lecanicillium lecanii* increased its virulence against the aphid *Aphis gossypii* when Pr1A was over-expressed as compared to the wild type [30]. These observations correlate with those of known subtilisin mutations (SNPs-based information) or gene loss and changes in virulence. Furthermore, spontaneous Pr1A- and Pr1B-deficient mutants of *M. anisopliae* showed significantly lower Pr1 and elastase activities and were less virulent towards the coleopteran *Tenebrio molitor*, but no change was observed for the lepidopteran *Galleria mellonella* [31].

No trypsin-like proteases were detected during six days of liquid culture by assaying the supernatant of our *B. bassiana* with Z-FR-pNA as substrate. This is in contrast with previous studies with *M. anisopliae* documenting that *Metarhizium* secretes trypsin-like proteases (e.g., genbank AAD29675, trypsin-like protease Pr2A, and BAB70707, elastase-like serine protease from *M. anisopliae* [32]). The trypsin like activity (i.e., leucine aminopeptidase, genbank EFZ03923; serine carboxypeptidase S28, EFY95167) found in *Metarhizium* sp. by proteomics [13] and by conventional chromatography [33] could contribute to the total proteolytic activity compensating the lower chymotrypsin-like activity compared to *B. bassiana*.

Even if a sequential action of exoenzymes is speculated, where lipase plays an initial role [9,10,11] the virulence of *B. bassiana* isolates seems to depend foremost on the capability to hyper produce both proteases and chitinases [12]. However, here we have shown that the lipase 1 (uniprot A0A0A2W0S0) secretion in *B. bassiana* isolates has a variable baseline of expression with the highest level only observed when the intact cockroach diet regime was used (in Bb813, Figure 2). The lipase 1 found in this study (uniprot A0A0A2W0S0) has not yet been characterized and has a low degree of homology with the recombinant triacylglycerol lipase A (uniprot J4UGV0) used for biodiesel production [15]. The epicuticular layer of insects consists of a complex mixture of non-polar lipids including hydrocarbons, fatty acids, and wax esters, and the sets of lipid-degrading enzymes detected here (lipase 1 and other minor lipolytic esterases, Table 2) may have a dominant role in the breakdown of the epicuticle. The lipase 1 probably possesses broad esterase activity and could be able to generate alkanes and fatty acids as breakdown products that eventually will be used by the fungi as a carbon source using a specific subset of fungal cytochrome P450 monooxygenases involved in insect hydrocarbon degradation [34].

In *B. bassiana* the lipolytic activity was proposed as a ‘virulence index’, contributing to the evolution of host-parasite relationship, contributing to the selection for efficient isolates in order to adapt to the insect diversity [12,35].

In *M. anisopliae* the in vitro production and assay of cuticle-degrading enzyme activities, such as for chitinase, proteinase, lipase, and amylase in fourteen isolates exhibited significant natural isolate variability [36]. Similarly, the variation of the amount of the exoenzymes produced by *B. bassiana* in our study could reflect an adaptive host pathogenicity and diet specificity that could be investigated in future experiments.

Chitin represents the second most abundant polymer in insect cuticle. *B. bassiana* possesses twenty GH18 chitinases, the highest number among fungi when compared to plant pathogens (which on average possess 11 GH18 enzymes) [37]. Fungal chitinases are subdivided into four subgroups A, B, C, and D: (A) characterized by the absence of a chitin-binding domain, CBM; (B) having only one CBM at the C-terminal; (C) possessing two specific CBMs, CBM18 and CBM50 (LysM chitin-binding modules) and (D) having one CBM18 and a transglycosylation (TG) domain like in *Serratia* sp. or *Bacillus* sp.

Since many chitinases in entomopathogenic fungi are often under catabolic repression, their secretion prior to host invasion has to be continuously induced [38,39] by medium containing excess of chitin as substrate, as done here using 1% shrimp chitin as inducer.

We were able to detect an endo-chitinase D (A0A0A2VG14) as main chitinase differentially expressed more on intact insect diet in Bb 813. Actually, when compared to isolated cuticle diet in Bb813, it increased almost twice as much (Table 2), but chitinase D was also expressed in high amounts in Bb 949 using isolated cuticle nutrition, showing a high degree of variability among the isolates. Over-expression of Bbchit1 chitinase (genbank nucleotide seq. AY145440, protein AAN41259) under a constitutive promoter has been shown to enhance the virulence of *B. bassiana* to aphids [33,40]. Endo-chitinase D (A0A0A2VG14) and Bbchit1 chitinase are 99% identical.

Two other endo-chitinases (A0A0A2VB74 and A0A0A2V532) were only detected using the isolated cuticle diet in Bb 813 (Table 1). Together with an endo chitosanase (GH75, A0A0A2VUK5, Table 1) these two endo-chitinases were filtered out by the software algorithm of Progenesis QI for proteomics due to their low abundance in the rest of the cases. Nevertheless, they might also represent important chitinases involved in *B. bassiana* virulence, the recovery of which from the culture media may have been adversely affected by being strongly bound to the chitin and hence having poor solubility. 

We also detected two secreted LysM (pfam PF01476) proteins in *B. bassiana*, one of which (A0A0A2VL54, Table 2) possesses five lysine-containing motifs within the enzyme involved in binding, in this case, not of chitin, but of bacterial peptidoglycan. This autolysin is responsible for bacterial lysis since enzymes possessing multiple LysM motifs are associated with *N*-acetylmuramoyl-l-alanine amidase (autolysin) activity against the bacterial cell wall [41]. The other LysM-containing proteins (A0A0A2V6S0) possess two LysM motifs more similar to CMB50 with chitin-binding ability and are similar to the effector protein Ecp6 of the fungal plant pathogen *Cladosporium fulvum* (uniprot B3VBK9). Microbial pathogens secrete effector proteins to suppress PAMP (Pathogen-Associated Molecular Pattern)-triggered host immunity in order to establish infection. The LysM domain (CBM50) of Ecp6 mediates virulence through perturbation of chitin-triggered host immunity [42]. The perturbation by this LysM domain is not mediated through chitin sequestration or masking but possibly through interference with the host immune receptor complex, since LysM effectors outcompete plant host receptors for chitin binding [43]. Different entomopathogenic fungi like *B. bassiana* are also plant endophytes. In *B. bassiana* the LysM-containing proteins (A0A0A2V6S0) might be involved in the recognition of the endophytic phase for target plants [44,45]. In plants the LysM-containing proteins have been found in pattern recognition receptors (PRRs) that enable the plant to identify microbial symbiotic partners or pathogens. In *B. bassiana* the LysM protein (A0A0A2V6S0), homologue of some *Piriformospora indica* LysM proteins, could also inhibit chitin oligosaccharide-triggered and PRR-mediated activation of host immunity and establish root symbiont mutualistic relationships [45,46,47]. 

From the host immune response point of view [48], in particular in the insect cuticles and in the hemolymph, protease inhibitors involved in proteolytic immune cascades are present, the so-called serpins [49]. The insect´s serpin is a multigene family with many isogenes/isoforms in order to inhibit both bacterial and fungal proteases, including subtilisins (i.e., *M. anisopliae* Pr1A) [50]. One of the serpin variants, serpin-1J, strongly inhibited the activation of *Manduca sexta* hemolymph phenoloxidase, whose activation is achieved by a serine proteinase cascade. This pathway is a component of the defensive response of insects to microbial/fungal infection. Phenoloxidase catalyzes reactions that produce quinolic substances that either cross-link proteins and chitins in cuticular sclerotization or polymerize to form melanin [48]. Entomopathogenic fungi and their insect hosts evolve in a parallel way to adapt to the host, and vice versa the host tries to escape from being prey, and this phenomenon is well described at the level of co-evolution of fungal proteinases and corresponding host-derived proteinase inhibitors by the appearance of single nucleotide polymormisms (SNPs) that cause amino acid (AA) changes in both proteinase and inhibitors [51].

## 5. Conclusions

In conclusion, our study represents the first report of a detailed up-to-date proteomics study on *B. bassiana* exoenzymes from several isolates elucidating their quantitative and qualitative aspects. Previously, *B. bassiana* exoenzymes were only reported as enzyme activities or partially purified activities from extracellular artificial media containing intact insect or crushed/purified cuticles. Here we describe a precise quantitative/qualitative secretome of several fungal isolates according to the nutrition based on either intact dead cockroach diet or isolated cuticle diet.

These exoenzymes, produced as recombinant enzymes (recombinant fungus-free bio-insecticides), could potentially overcome the endogenous inhibitors in insects, when used in high amounts. Spray formulation of such complex mixtures (e.g., lipase 1, Pr1A, Pr1B, Smp1, and Chitinase D) could maybe increase their bioinsecticidal efficacy by reducing the killing time as compared to the entomopathogenic fungi per se.

## Figures and Tables

**Table 1:** Qualitative proteomics identification of *B. bassiana* exoenzymes/proteins present on day four in the supernatant of isolate 813 grown in the presence of intact cockroaches. Protein hits were ordered based on their peptide number and abundance (spectral counting) as obtained by PLGS 2.5 software (Waters, Milford, MA, USA). Annotation from Uniprot website cross-checked within several protein domain database websites (InterPro, https://www.ebi.ac.uk/interpro/; Genbank, https://www.ncbi.nlm.nih.gov/genbank/; a.o.).

Accession	Description	mW (Da)	Peptides	Coverage (%)
A0A0A2W0R3	Pr1A Alkaline serine protease	38,793	12	38.2586
A0A0A2VW01	Pr1B Alkaline protease 1	41,528	11	27.8481
A0A0A2VRL8	l-ascorbate peroxidase	101,456	8	10.9959
A0A0A2VJ73	Alpha galactosidase	57,651	7	12.6629
A0A0A2VDM1	Putative glycosidase crf1	38,406	6	17.4142
A0A0A2VIV9	Isochorismatase-like (Cysteine hydrolases) protein	25,783	5	18.8034
A0A0A2VJP2	ATP dependent RNA helicase glh 2	51,230	5	11.8068
A0A0A2VQS8	Cell_wall_mannoprotein_1	19,361	5	22.4599
A0A0A2V7C2	Beta-1,3 exoglucanase (Carbohydrate-binding WSC domain)	18,985	4	21.5909
A0A0A2V928	Catalase peroxidase	87,240	4	3.7641
A0A0A2VPJ3	Extracellular aldono-Lactonase	41,232	4	10.4859
A0A0A2VSY1	Subtilisin like proteinase Spm1	56,909	4	6.5789
A0A0A2VUK5	Endo chitosanase	31,664	4	14.6667
A0A0A2V8V1	Vacuolar protease A	42,377	3	7.0886
A0A0A2VHX5	Six-hairpin glycosidase GH13	98,237	3	4.6804
A0A0A2VL54	Autolysin (Lysine Motif, LysM domain-containing protein)	44,154	3	6.5375
A0A0A2VR76	Carboxypeptidase Y A	97,773	3	2.4719
A0A0A2V6S0	LysM motifs (double) protein	49,033	2	3.9735
A0A0A2VB74	Endo beta N acetylglucosaminidase F2	71,647	2	3.0722
A0A0A2VDW0	Concanavalin A-like lectin/glucanases superfamily	21,878	2	8.7379
A0A0A2VFU8	Thioredoxin reductase	46,622	2	4.1475
A0A0A2VG14	Chitinase D	34,889	2	8.7879
A0A0A2VKM6	Metallo-Zn-Carboxypeptidase A-like protein (M14A)	43,858	2	5.7789
A0A0A2VMQ3	Fluoride ion transporter	7335	2	25
A0A0A2VUX9	Putative dipeptidyl peptidase 5	109,325	2	2.0121
A0A0A2VWX1	Alpha N arabinofuranosidase	53,574	2	4.3738
A0A0A2VZ64	Glucan 1,3 beta glucosidase	83,915	2	3.0968
A0A0A2W0E3	Tripeptidyl peptidase sed2	59,491	2	4.6181
A0A0A2W0S0	Lipase 1	51,056	2	4.7228
A0A0A2V532	Chitinase, Glycosyl hydrolase 18 family (GH18)	39,980	1	3.0137
A0A0A2VAD7	Small secreted protein	14,925	1	7.8571
A0A0A2VC64	Glutathione reductase	51,184	1	2.537
A0A0A2VCD5	Transcription factor Opi1	24,366	1	4.2735
A0A0A2VCZ9	Neutral cholesterol ester hydrolase	36,385	1	2.1407
A0A0A2VDJ2	Hypoxia up-regulated protein 1	112,148	1	1.564
A0A0A2VES3	Regulation of enolase protein 1 (DUF1349)	20,815	1	5.8201
A0A0A2VFI0	Cytochrome oxidase assembly protein 1	24,527	1	4.5872
A0A0A2VFS0	Dolichyl phosphate mannose protein mannosyltransferase 1	100,507	1	0.5543
A0A0A2VGF4	Putative serine protease S8 K12H47-like	57,301	1	2.3438
A0A0A2VJQ2	Assimilatory nitrite reductase (NirD) small subunit	8982	1	13.6364
A0A0A2VM88	Putative alpha beta glucosidase agdC	54,004	1	2.459
A0A0A2VMZ6	RNA pyrophosphohydrolase (Nudix)	18,709	1	8.7209
A0A0A2VR04	Putative 60S ribosomal protein MRP49	22,558	1	3.9216
A0A0A2VRG8	Putative U3 small nucleolar RNA-associated protein 13	166,473	1	0.3979
A0A0A2VW39	tRNA Guanine 37 N1 methyltransferase	28,437	1	1.9608
A0A0A2W0I5	Pre rRNA processing protein esf1	84,406	1	1.7196
A0A0A2W3A3	Beta glucosidase	94,734	1	1.6018
A0A0A2W457	Glycerol 3 phosphate dehydrogenase	56,490	1	1.5936
A0A0A2W4E7	Ribose import ATP binding protein RbsA	46,279	1	1.6667
A0A0A2WIE5	Aspartic protease	37,508	1	2.5352

**Table 2:** Absolute quantification of differentially secreted proteins from *B. bassiana* isolates under different growth conditions in liquid medium. Isolate Bb 813 was grown both in intact cockroach medium (“intact”) and in isolated cuticle medium (“cuticle”) used also for the other isolates. Progenesis QI for proteomics results have been ENO1 normalized and proteins sorted by their absolute amount calculated in fmol/µL.

Accession	Peptide Counts	ANOVA (p)	Description	Intact		Cuticle		Cuticle		Cuticle		Cuticle		Cuticle		Cuticle	
				Bb 813	Stdev	Bb 813	Stdev	Bb BG	Stdev	Bb 949	Stdev	Bb 893	Stdev	Bb 715	Stdev	Bb 678	Stdev
A0A0A2VJD2	2	2.11E-15	unknown protein	508.94	48.82	81.64	3.57	57.58	1.36	42.54	1.64	34.23	3.94	28.18	3.66	18.11	2.98
A0A0A2W0R3	23	0	Pr1A Alkaline serine protease	267.04	31.07	41.88	2.67	99.87	0.89	52.44	0.27	20.05	0.85	45	1.09	62.18	0.65
A0A0A2VQS8	40	0	Cell wall mannoprotein 1	241.81	3.24	739.44	12.22	115.12	2.38	217.32	3.23	80.16	0.48	45.72	2.44	45.13	1.08
A0A0A2VHX5	8	1.11E-16	Six-hairpin alpha-glycosidase GH13	167.49	9.82	33.39	0.39	64.16	2.01	47.65	1.94	26.81	0.48	28.57	1.82	44.26	0.95
P00924	41	1.57E-13	Enolase 1 (*Saccharomyces cerevisiae*)	100	1.43	100	3.02	100	2.68	100	1.57	100	2.33	100	9.12	100	3.61
A0A0A2VW01	15	1.96E-12	Pr1B Alkaline protease 1	81.89	4.07	96.01	2.03	70.87	1.34	88.23	1.74	62.95	1.6	32.48	2.63	43.25	1.34
A0A0A2VG14	10	0	Chitinase D	75.71	2.34	48.53	0.69	48.46	0.9	68.41	1.54	47.39	0.22	13.82	0.98	14.18	0.67
A0A0A2VDM1	8	9.23E-10	Putative glycosidase crf1	60.45	1.49	131.68	0.38	115.36	4.46	104.15	1.18	97.46	4.35	69.12	9.38	74.13	4.04
A0A0A2VPJ3	17	8.69E-13	Extracellular aldono-Lactonase YkgB	48.86	2.38	36.56	0.94	98.42	4.14	100.11	0.49	57.65	0.36	46.46	3.42	43.88	1.93
A0A0A2VRL8	17	1.68E-13	l-ascorbate peroxidase	43.14	0.62	95.94	3.58	198.51	3	180.37	3.08	71	0.82	49.6	5.06	65.99	1.46
A0A0A2W9X8	5	8.81E-09	Sialidase	42.84	8.4	12.39	0.51	15.17	0.85	16.26	2.72	9.5	1.15	12.28	0.68	17.55	0.73
A0A0A2VIV9	5	0	Isochorismatase-like (Cysteine hydrolases) protein ycaC	39.05	2.27	3.32	0.44	2.12	0.12	1.5	0.09	0.84	0.06	0.61	0.05	0.91	0.07
A0A0A2VL34	2	0	Putative glucan endo-1,3-beta-glucosidase eglC	37.09	0.84	9.07	0.32	7.54	0.22	14.27	0.39	6.76	0.21	1.4	0.18	1.69	0.09
A0A0A2VJ73	11	1.69E-13	Alpha-galactosidase	35.98	3.93	28.71	0.91	7	0.58	12.49	0.18	10.14	0.71	2.73	0.76	3.27	0.18
A0A0A2VSY1	2	0	Subtilisin-like proteinase Spm1	35.86	1.1	4.14	0.28	6.74	0.32	4.84	0.02	3.02	0.1	2.6	0.21	3.13	0.16
A0A0A2VGF4	5	6.83E-10	Putative serine protease S28 (K12H4.7)	30.8	1.58	21.24	1.24	26.17	1.03	23.87	0.15	12.15	0.34	12.71	1.52	16.64	0.6
A0A0A2W0S0	2	0	Lipase 1	28.66	0.88	6.82	0.21	2.74	0.06	2.83	0.06	1.99	0.2	1.31	0.16	2.12	0.07
A0A0A2V928	13	2.01E-12	Catalase-peroxidase	28.1	0.97	13.73	0.6	20.14	0.04	27.45	1.42	13.54	0.81	10.64	0.2	13.91	0.26
A0A0A2VUK5	2	3.44E-15	Endo-chitosanase	27.53	0.56	20.96	1.29	2.81	0.1	3.18	0.18	1.84	0.24	1.08	0.26	2.24	0.36
A0A0A2VL54	4	2.22E-16	Autolysin (Lysine Motif, LysM protein)	25.57	1.91	19.84	0.53	26.24	0.88	24.45	0.94	15.83	0.95	71.63	5.57	59.59	2.1
A0A0A2V8V1	3	6.66E-16	Vacuolar protease A	25.04	0.54	10.77	0.61	5.58	0.25	7.29	0.01	4.8	0.23	2.74	0.26	4.18	0.19
A0A0A2VC64	1	1.11E-15	Glutathione reductase	24.96	1.19	17.78	0.77	43.06	1.81	29.62	0.36	13.94	0.97	41.15	4.7	89.06	4.45
A0A0A2VWW0	6	1.17E-13	Cell_wall_mannoprotein_1	23.67	1.06	26.27	0.91	28.03	0.25	45.77	1.2	17.12	0.08	32.77	2.23	24.19	0.3
A0A0A2V7C2	6	9.10E-15	Beta-1,3 exoglucanase	22.11	1.2	12.72	0.16	11.1	0.09	19.31	0.73	12.48	0.49	9.91	0.22	11.66	0.33
A0A0A2VMQ3	1	0.02458	Fluoride ion transporter	19.72	1.12	8.4	3.16	3.8	0.98	7.24	3.82	4.53	3.42	6.5	2.18	6.17	1.31
A0A0A2VJ18	10	7.17E-14	Reticulocyte-binding protein 2 a	18.09	0.33	63.6	1.29	74.32	1.83	65.55	4.05	63.73	5.35	52.53	1.55	58.97	1.71
A0A0A2VAM6	1	2.81E-14	Putative glucan endo-1,3-beta-glucosidase eglC	16.03	0.59	7.76	0.33	9.88	0.24	8.71	0.11	2.39	0.48	2.82	0.17	1.54	0.06
A0A0A2W3A3	5	5.38E-13	Beta-glucosidase	15.3	1.67	130.33	11.33	27.68	1.13	35.33	0.44	18.32	0.84	14.95	2.1	12.71	0.34
A0A0A2VR76	4	2.29E-12	Carboxypeptidase Y A	13.82	1.27	9.87	0.29	27.52	1.49	17.53	0.29	8.67	0.48	20.36	1.58	10.61	0.34
A0A0A2VFU8	5	9.04E-11	Thioredoxin reductase	12.18	0.89	7.9	0.16	13.3	0.23	7.96	0.69	3.68	0.23	7.42	1.02	10.84	0.29
A0A0A2VWX1	4	5.55E-16	Alpha-*N*-arabinofuranosidase	12.01	0.51	14.49	0.3	72.11	1.86	38.57	0.78	19.18	0.15	11.18	0.93	16.19	0.2
A0A0A2VZS6	2	2.69E-06	Putative J domain-containing protein C3E7.11 c	11.57	0.1	10	0.29	14.16	0.55	11.2	1.71	8.42	0.24	9.66	0.63	11.22	0.41
A0A0A2VS40	2	4.04E-10	Protein NIF3 (NGG1p interacting factor 3)	10.84	3.32	3.86	0.27	5.43	0.35	15.87	0.48	5.62	0.37	1.94	0.34	0.98	0.3
A0A0A2VNH3	3	2.92E-13	Beta-galactosidase	10.83	0.49	13.32	0.9	59.3	1.86	33.18	0.48	16.36	0.76	8.17	1.06	7.79	0.38
A0A0A2VSI9	1	1.12E-12	Putative Zn(II)2Cys6 transcription factor	10.63	0.76	6.31	0.23	5.55	0.38	8.48	0.05	5.34	0.36	7.65	0.36	8.82	0.42
A0A0A2VWS4	11	3.11E-15	Alkaline phosphatase H	10.34	0.45	21.51	0.14	41.28	1.21	22.39	0.69	20.16	0.36	34.08	4	36.46	1.44
A0A0A2VAJ1	8	1.10E-09	Tripeptidyl-peptidase sed1	9.78	0.66	9.15	0.45	12.12	0.5	6.77	0.1	5.5	0.29	4.92	0.47	5.14	0.22
A0A0A2VMK7	2	1.92E-14	AP-1-like transcription factor	8.88	0.43	5.01	0.32	50.81	2.31	22.31	0.12	8.58	0.56	4.09	0.59	7.17	0.36
A0A0A2VGH0	2	1.43E-08	Glucan endo-1,3-beta-glucosidase	7.38	0.82	7.2	0.35	7.87	0.32	7.2	0.15	5.65	0.58	8.21	1.52	7.21	0.64
A0A0A2VJP2	2	1.31E-07	ATP-dependent RNA helicase glh-2	7.14	1.24	11.55	0.36	8.05	0.23	9.7	0.18	8.44	0.3	6.39	0.73	6.22	0.22
A0A0A2VZ64	2	3.61E-07	Glucan 1,3-beta-glucosidase	6.85	0.98	7.54	0.38	9.81	0.6	7.66	0.15	5.3	0.33	5.9	0.53	8.29	0.19
A0A0A2VUJ3	3	0	Glutamine-tRNA ligase	6.45	0.17	6.36	0.24	11.56	0.48	22.08	0.27	10.71	0.38	48.56	5.78	44.18	1.51
A0A0A2W0E3	5	1.69E-09	Tripeptidyl-peptidase sed2	4.31	0.23	15.1	0.84	27.45	1.2	12	1.75	5.68	0.92	8.26	0.88	19.98	0.48
A0A0A2VBW0	2	5.20E-05	Translation initiation factor IF-2	3.39	0.2	18.95	3.7	61.44	13.35	31.76	10.98	11.81	6.98	17.17	1.78	35.49	1.88
A0A0A2V6D8	4	3.54E-14	Flagellar motor protein MotB (i.e., XP_007808661)	2.82	0.45	6.27	0.38	35.33	1.72	13.78	0.1	8.35	0.81	25.02	3.04	13.94	0.84
A0A0A2V5R3	2	0	Glycosyltransferase family 90 protein	2.2	0.17	6.55	0.22	3.82	0.25	37.63	0.58	40.78	2.24	4.29	0.33	3.43	0.11
A0A0A2VPN1	1	9.31E-12	BTB/POZ domain zinc finger transcription factor	1.12	0.46	14.77	0.58	4.1	0.58	7.31	0.3	4.61	0.11	0.14	0.03	2.15	0.16
A0A0A2W8Q3	1	8.40E-12	Brefeldin A resistance protein	1.06	0.35	4.07	0.36	2.99	0.16	7.92	0.24	3.08	0.5	9.9	1.32	8.06	0.66
A0A0A2VIC5	1	4.44E-16	Nickel/cobalt efflux system rcnA	1.01	0.22	2.16	0.2	1.14	0.07	2.25	0.16	0.01	0.01	12.33	1.21	5.93	0.24
A0A0A2W2R1	1	4.33E-15	Glucan synthesis regulatory protein	0.83	0.04	2.27	0.27	25.72	1.04	14.57	0.55	4.46	0.15	1.55	0.25	2.58	0.24
A0A0A2VD91	2	1.27E-14	alpha-1,2-Mannosidase	0.01	0	1.86	0.12	1.21	0.03	1.43	0.17	0.06	0.02	7.07	1.57	6.32	1.35
0A0A2VEQ5	1	0	DNA polymerase III subunits gamma/tau	0	0	0.53	0.08	0.62	0.09	0.63	0.07	0.01	0.01	5.61	0.74	9.17	0.13
A0A0A2VWT7	3	0	Phospholipase A2	0.18	0.02	4.35	0.27	15.73	0.7	11.09	0.09	7.21	0.29	8.59	0.91	3.32	0.26
